# Luteolin Inhibits Invasion of *Listeria monocytogenes* by Interacting with SortaseA and InternalinB

**DOI:** 10.3390/molecules31020297

**Published:** 2026-01-14

**Authors:** Junlu Liu, Rui Liu, Hang Pan, Jiahui Lu, Qiong Liu, Guizhen Wang

**Affiliations:** 1 College of Biological and Food Engineering, Jilin Engineering Normal University, Changchun 130052, China; liujunlu@stu.jlenu.edu.cn (J.L.); liurui@stu.jlenu.edu.cn (R.L.); panhang@stu.jlenu.edu.cn (H.P.); lujiahui@stu.jlenu.edu.cn (J.L.); liuqiong@jlenu.edu.cn (Q.L.); 2 Engineering Research Center of Microecological Vaccines (Drugs) for Major Animal Diseases, Ministry of Education, Jilin Agricultural University, Changchun 130118, China

**Keywords:** internalins, SortaseA, *Listeria monocytogenes*, Luteolin, anti-infection

## Abstract

*Listeria monocytogenes* (LM) is a lethal foodborne intracellular pathogen. Internalins A and B (inlA and inlB) are critical virulence factors that promote LM’s adhesion and invasion into host cells. InlA is covalently anchored to the cell wall by LM SortaseA (SrtA), while inlB is anchored to the cell wall via non-covalent bonds. Therefore, inhibiting SrtA and inlB is expected to suppress LM’s adhesion and invasion of host cells, enabling the prevention and control of infections. This study demonstrated that Luteolin inhibited the activity of purified LM SrtA protein in vitro. Interactive mechanism analysis indicated that Luteolin generates interaction with the critical active sites of SrtA, which may affect its binding to its natural substrates, thereby reducing the anchoring of inlA on the cell wall and achieving the inhibition of bacterial adhesion and invasion. In addition, Luteolin binds to the groove at the binding interface between inlB and its host receptor. The key residues in inlB that interact with the host receptor form weak interactions (Hydrogen bonds and van der Waals interactions) with Luteolin, this binding may inhibit their binding, suppressing LM’s adhesion and invasion of host cells. At the tested concentrations, Luteolin did not affect the growth of LM, but remarkably reduced the mortality and alleviated the infection symptoms of LM-infected *Galleria mellonella*. These results provide additional theoretical evidence for the application of Luteolin in the prevention and control of LM infections, which is expected to accelerate its application progress.

## 1. Introduction

*Listeria monocytogenes* (LM) is a significant zoonotic and fatal foodborne pathogen [1,2]. It can cause infections in humans, livestock, and poultry, with a mortality as high as 30% [3]. LM can survive continuously under low-temperature conditions, and people can be infected by contaminated food [2]. LM is an intracellular bacterium that can cause meningitis and fetal abortion through the blood–brain barrier and placental barrier system under a multiple toxin synergistic reaction [4]. The currently severe situations of bacterial drug resistance make the prevention and control of LM encounter challenges [5]. Developing natural compounds from Chinese herbal plants to combat LM infection is an alternative strategy, and some positive progresses have been obtained [6].

**Scheme 1:**
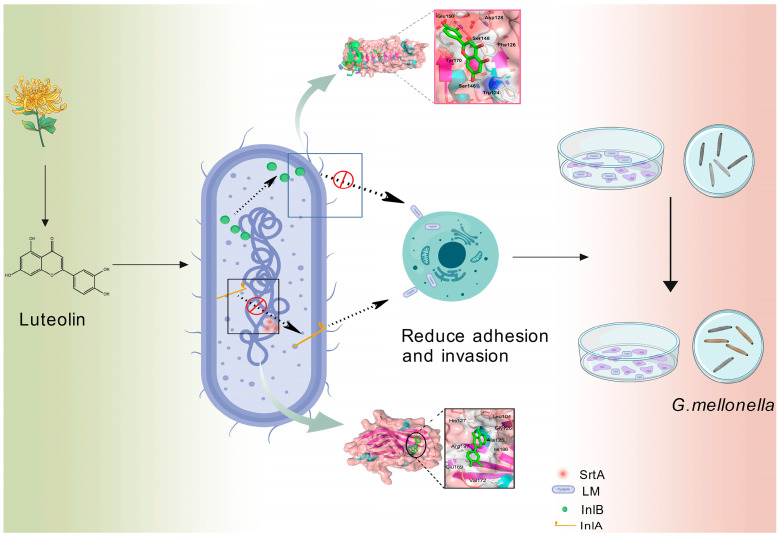
Schematic illustration of Luteolin against LM infection by inhibiting SrtA and InlB. Luteolin interacts with SrtA and InlB to reduce the adhesion and invasion of LM to the host cells, then Luteolin protects *G. mellonella* from LM infection.

As an intracellular bacterium, adhering and invading into the host cells is critical for the bacteria to achieve a successful infection [7]. LM internalin proteins play a significant role in promoting LM adhesion and invasion [8]. The internalin family of LM comprises over twenty member proteins, but internalin A (inlA) and internalin B (inlB) are the most important members on promoting the adhesion and invasion of LM [9,10]. InlA contains an LPXTG motif which can be recognized by LM SortaseA (SrtA); then, SrtA cleaves the LPXTG motif between T and G and then inlA is covalently anchored to the bacterial cytoderm [11]. InlB was anchored to the cell wall by non-covalent bonds. InlA interacts with e-cadherin and inlB interacts with tyrosine kinase, these interactions help LM adhere and invade host cells to trigger diseases [12,13,14]. It has been demonstrated that the adhesion and invasion of LM to host cells were significantly reduced when inlA and inlB were knocked out, resulting in lower pathogenicity on experimental animal models [15,16]. These reports suggest that inlA and inlB are important for LM infection. The Luteolin is a natural flavonoid and it was widely found in many plants [17], such as *Platycodon grandiflorum* [18], *Gentianopsis paludosa* [19], *Ajuga decumbus* [20], *Lonicera japonica Thunb* [21], and so on. Luteolin has been reported as an anticancer agent [22,23] and a modulator of skin aging and inflammation [24]. In addition, it has also been reported to inhibit Alzheimer’s disease [25]. Wang et al. [26] found that Luteolin inhibits the expression and secretion of listeriolysin O (LLO), which is a key virulence factor of LM. However, Luteolin targets inlA and inlB, or SrtA and inlB, to suppress the adhesion and invasion of LM, have not been reported. This work aims to explore natural compounds that inhibit adhesion and invasion by targeting virulence factors and to provide new insights into non-antibiotic strategies for controlling LM infection. It was found that Luteolin binds to the active center of LM SrtA and interacts with the active sites residues to reduce its transpeptidase activity, which results in a reduction in inlA anchoring to the cell wall. In addition, Luteolin also generated interactions with the residues of inlB that are participating in the binding to its host receptor. These interactions result in a significant decrease in LM adhesion and invasion to host cells. Luteolin did not show anti-LM character under the tested concentrations, but it protected *Galleria mellonella* from LM infection. Our result will provide more theoretical evidence for Luteolin being used against LM infection (Scheme 1).

## 2. Results

### 2.1. Analysis of the Results

#### 2.1.1. Luteolin Inhibits the Transpeptidase Activity of LM SrtA by Binding to the Active Center

**Figure 1:**
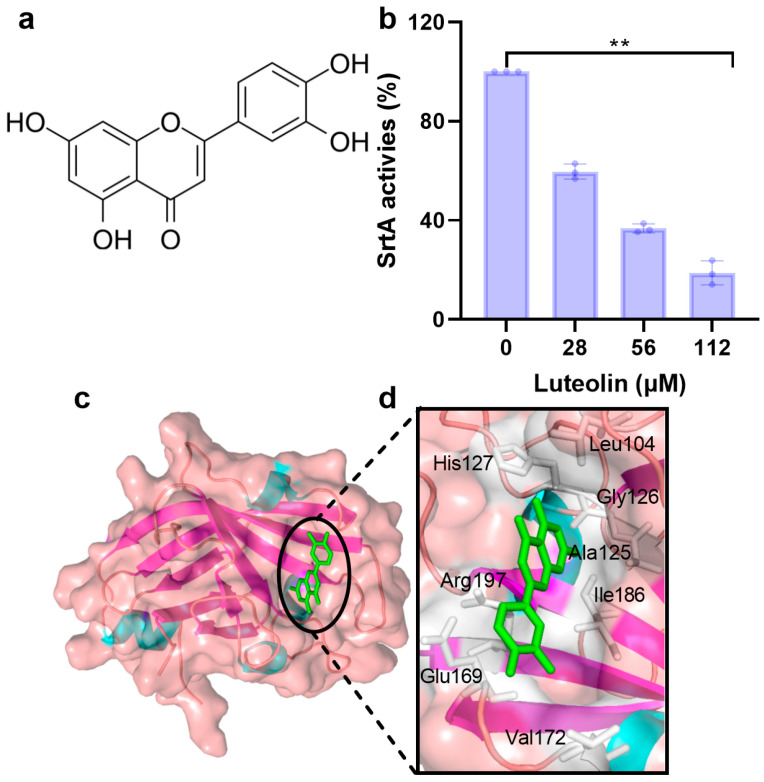
Luteolin inhibits the transpeptidase activity of LM SrtA by binding to the active center. (**a**) The molecular structure of Luteolin. (**b**) The inhibitory effect of Luteolin against SrtA transpeptidase activity. Data are presented as means ± SDs, n = 3, ** indicating *p* < 0.01. (**c**,**d**) The binding mode and potential binding sites between Luteolin and SrtA. The protein was presented as surface with the transparency of 50% and colored by secondary structures. Luteolin was shown as stick and colored in green.

The molecular structure of Luteolin (Purechem-Standard Co., Ltd., Chengdu, China) is shown in Figure 1a. To evaluate the inhibitory effect of Luteolin on the activity of LM SrtA, we synthesized substrate peptides with fluorescent groups at both ends and conducted SrtA activity inhibition experiments. The results showed that when no Luteolin was presented in the reaction system, strong fluorescence signals were detected at the reaction endpoint, with the SrtA activity defined as 100%. When different concentrations of Luteolin were added to the reaction system, the relative activity of SrtA gradually decreased (Figure 1b), indicating that Luteolin inhibits the activity of LM SrtA in a concentration-dependent manner, with the half maximal inhibitory concentration (IC_50_) of 37.47 µM. This result also indicates a direct binding exists between Luteolin and SrtA; for confirmation, molecular docking calculation was performed. It was found that Luteolin bound to the active center of SrtA and some residues generated interaction with Luteolin, mainly including His127, Arg197, Ile186, Leu104, and so on (Figure 1c,d), among which His127 and Arg197 are the critical residues for the transpeptide activity of LM SrtA [27]. Therefore, these results indicate that Luteolin interacts with the active center of SrtA to inhibit its transpeptidase activity.

#### 2.1.2. Luteolin Interacts with the Active Sites Residues of SrtA to Inhibit Its Activity

**Figure 2:**
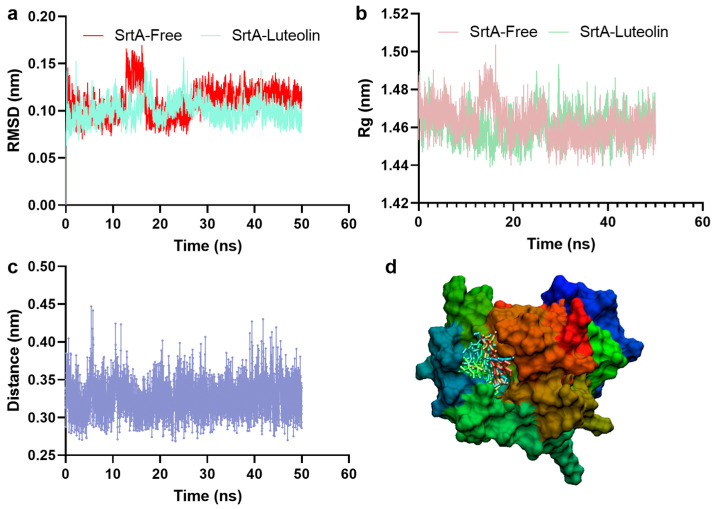
Luteolin and SrtA maintain stable binding. (**a**,**b**) The RMSD and Rg fluctuations of SrtA during the MD simulation. (**c**) The distance between Luteolin and SrtA. (**d**) The relative positions of Luteolin and SrtA during simulation. Luteolin and SrtA were colored by timesteps.

MD assay was carried out to confirm the binding sites between Luteolin and SrtA. The root mean square deviation (RMSD) fluctuations during the simulation process indicate that the protein maintains stable conformations when bound with or without Luteolin (Figure 2a), and the binding of Luteolin did not affect the structure of SrtA as the protein share similar radius of gyration (Rg) (Figure 2b). The distance between Luteolin and SrtA (Figure 2c) and the structural superposition of Luteolin and SrtA at different time points (Figure 2d) demonstrate that the binding between Luteolin and SrtA is reliable.

**Figure 3:**
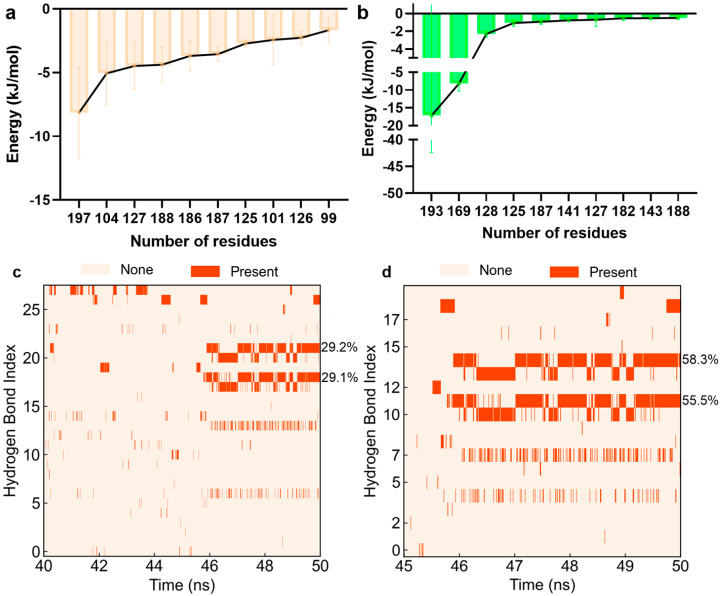
The weak interaction between Luteolin and SrtA. The residues in SrtA contribute higher electrostatic (**a**) and van der Waals (**b**) interactions to the binding. The Hydrogen bonds map between Luteolin and SrtA at 40–50 ns (**c**) or 45–50 ns (**d**).

Residue energy decomposition found that the Arg197, His127, Cys188, and Leu104 residues contribute more van der Waals (∆*G_vdw_*) interactions with the values of −8.16 kJ/mol, −4.48 kJ/mol, −4.38 kJ/mol, and −5.06 kJ/mol (Figure 3a), indicating that these residues form strong van der Waals interactions with Luteolin. Glu193, Glu169, and His128 provided more electrostatic interactions (∆*G_ele_*) with the values of −17.218 kJ/mol, −8.65 kJ/mol, −2.31 kJ/mol (Figure 3b), suggesting these residues promote the binding by generating electrostatic interaction. Though Hydrogen bonds were detected in the final 10 ns of the trajectory file, their occupancy was lower than 30% (Figure 3c). However, two pairs of Hydrogen bonds mainly appeared at the last 5 ns, with an occupancy of 58.3% and 55.5% (Figure 3d). Both of these two Hydrogen bonds existed between Glu193 and Luteolin, which resulted in higher electrostatic energy contribution of this residue. The smaller root mean square fluctuation (RMSF) values of these residues than those in the free protein also confirm the interaction (Figure S1). Arg197, His127, and Cys188 were identified as the key active sites of SrtA, as the transpeptidase activity of SrtA was almost completely lost when these residues mutated to Ala [27]. Here, Luteolin formed van der Waals interactions with Arg197, His127, and Cys188. These results indicate that Luteolin interacts with the critical active residues of SrtA to affect the binding between SrtA and its substrate.

For further confirmation, an enzyme kinetics experiment was performed; it was found that when a different content substrate peptide was treated with SrtA and Luteolin, the maximum reaction velocity (Vmax) did not show significant changes, but the Michaelis–Menten constant (Km) increased significantly (Figure S2a,b, Table S1), which indicates that Luteolin mainly acts as a competitive inhibitor of SrtA. These results correspond to the simulation results.

#### 2.1.3. Luteolin Does Not Affect LM Growth but Significantly Reduces Its Adhesion and Invasion to Host Cells

**Figure 4:**
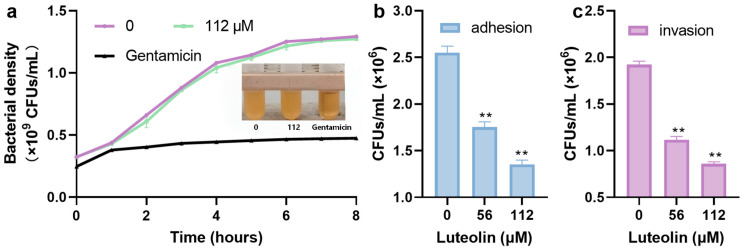
Luteolin does not affect LM growth but significantly reduces its adhesion and invasion to host cells. (**a**) The growth trends of LM when treated without or with Luteolin. Data were shown as means with SDs, n = 3. The adhesion (**b**) and invasion (**c**) of LM to host cells when treated with various concentrations of Luteolin. Data were shown as means with SDs, n = 3. ** represents *p* < 0.01.

The minimum inhibitory concentration (MIC) of Luteolin against LM is 448 μM (Table S2). The growth trends of LM were almost synchronous when LM co-cultured without or with Luteolin (Figure 4a), suggesting Luteolin does not exert growth stress to LM at the tested concentration. The lactate dehydrogenase (LDH) levels that were detected in the cultural supernatant of cells (Figure S3a) and the heme released to the medium (Figure S3b) evident Luteolin does not have cytotoxicity. Then, adhesion and invasion assay were performed and we found that the detected clones were 2.55 × 10^6^ colony forming units per milliliter (CFUs/mL) and 1.92 × 10^6^ CFUs/mL for adhesion and invasion; but, when samples received Luteolin treatment, the rate of adhesion decreased to 68.76% and 53.07% (Figure 4b), and for invasion, the data reduced to 58.06% and 44.71% (Figure 4c), indicating Luteolin decreases the adhesion and invasion of LM to host cells. InlA can be anchored to the cell surface by SrtA and promotes LM adherence and invasion to host cells. Luteolin inhibits the activity of SrtA, which may directly result in lower anchoring of inlA on the cell wall; thus, a reduction in adhesion and invasion of LM to host cells were observed.

#### 2.1.4. Luteolin Generates Weak Interactions with the Residues of inlB, Which Are Involved in the Binding with Host Receptor

**Figure 5:**
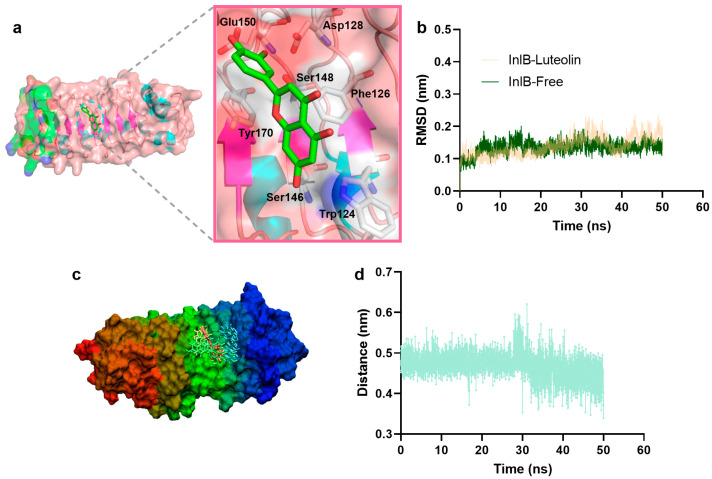
Luteolin binds to the groove of inlB. (**a**) The binding mode and potential binding sites of Luteolin and inlB. Luteolin was presented as stick and colored by element; inlB was shown as surface with the transparency of 40%. (**b**) The RMSD values of inlB that bound with or without Luteolin. (**c**) The relative position of Luteolin and inlB. InlB was shown as surface and colored by index, Luteolin was presented as licorice and colored by timesteps. (**d**) The distance between Luteolin and inlB during the MD assay.

The adhesion and invasion of LM to host cells are primarily mediated by inlA and inlB. To confirm whether Luteolin affects the function of inlB, we carried out a molecular docking experiment based on a X-ray structure of inlB that has been disclosed [28]. We found that Luteolin bound to the groove of inlB and interacted with residues of Glu150, Asp128, Ser148, and Tyr170 (Figure 5a). Then we aligned the configuration of Luteolin/inlB complex with inlB/receptor complex (PDB ID 6U12) to find that Luteolin bound to the junction of inlB and its host receptor (Figure S4).

We carried out a MD assay to confirm the reliability of the binding. The RMSD fluctuation of inlB (free or bound with Luteolin) (Figure 5b), the structural superposition of Luteolin and inlB at different times of the simulation (Figure 5c), and the distance between Luteolin and inlB during the simulation (Figure 5d) all indicate that Luteolin and inlB maintain a stable binding. The Rg values shown in Figure S5a indicate the binding of Luteolin did not affect the structure of inlB.

**Figure 6:**
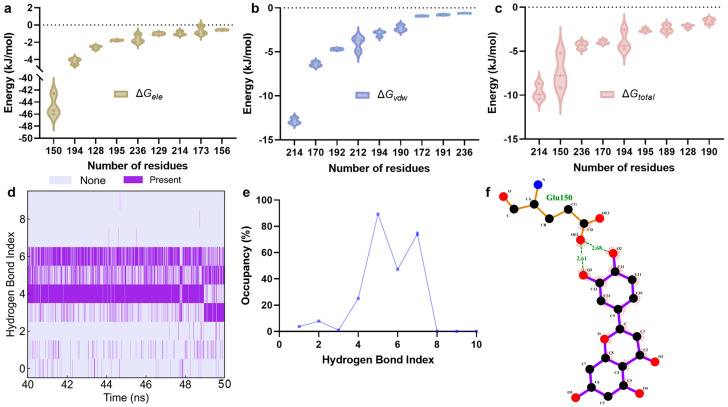
The residues in inlB that contribute binding free energies to the binding and the Hydrogen bonds generated between inlB and Luteolin. The residues that contribute electrostatic interactions (**a**), van der Waals interactions (**b**), and the total binding free energy (**c**) for the binding. Data are presented as means ± SDs, n = 3. (**d**) The Hydrogen bonds generated between Luteolin and inlB and their occupancy (**e**). (**f**) The atoms of inlB and Luteolin involved in the Hydrogen bonds interaction.

To determine the critical interactive residues, we performed residue energy decomposition; it was found that Glu150, Glu194, and Asp128 contributed more electrostatic interactions with the values of −45.39, −4.58, and −2.81 kJ/mol (Figure 6a). Tyr214, Tyr170, Glu194, and Glu236 contributed more van der Waals interaction energy (Figure 6b); therefore, the total energy contribution of these residues is also higher (Figure 6c). However, Asp189 had a high total binding free energy contribution; but the energy contribution of this residue was not detected in the electrostatic interaction and van der Waals interaction. After analysis, it was found that Asp189 contributed more solvation energy (−2.87 kJ/mol), suggesting that Asp189 promoted the binding through solvation.

As Glu150 contributed a higher electrostatic interaction, Hydrogen bond interaction was speculated to exist, so Hydrogen bond analysis was carried out. The results showed that Glu150 and Luteolin formed two pairs of stable Hydrogen bonds in the last 10 ns of the equilibrium trajectory (Figure 6d), with occupancy rates of 89.4% and 74.3% (Figure 6e), respectively. These Hydrogen bonds exist between atom OE3 of Glu150 and O2–O3 of Luteolin (Figure 6f). The RMSFs of these residues in the complex system were smaller than those in the free protein (Figure S5b).

**Figure 7:**
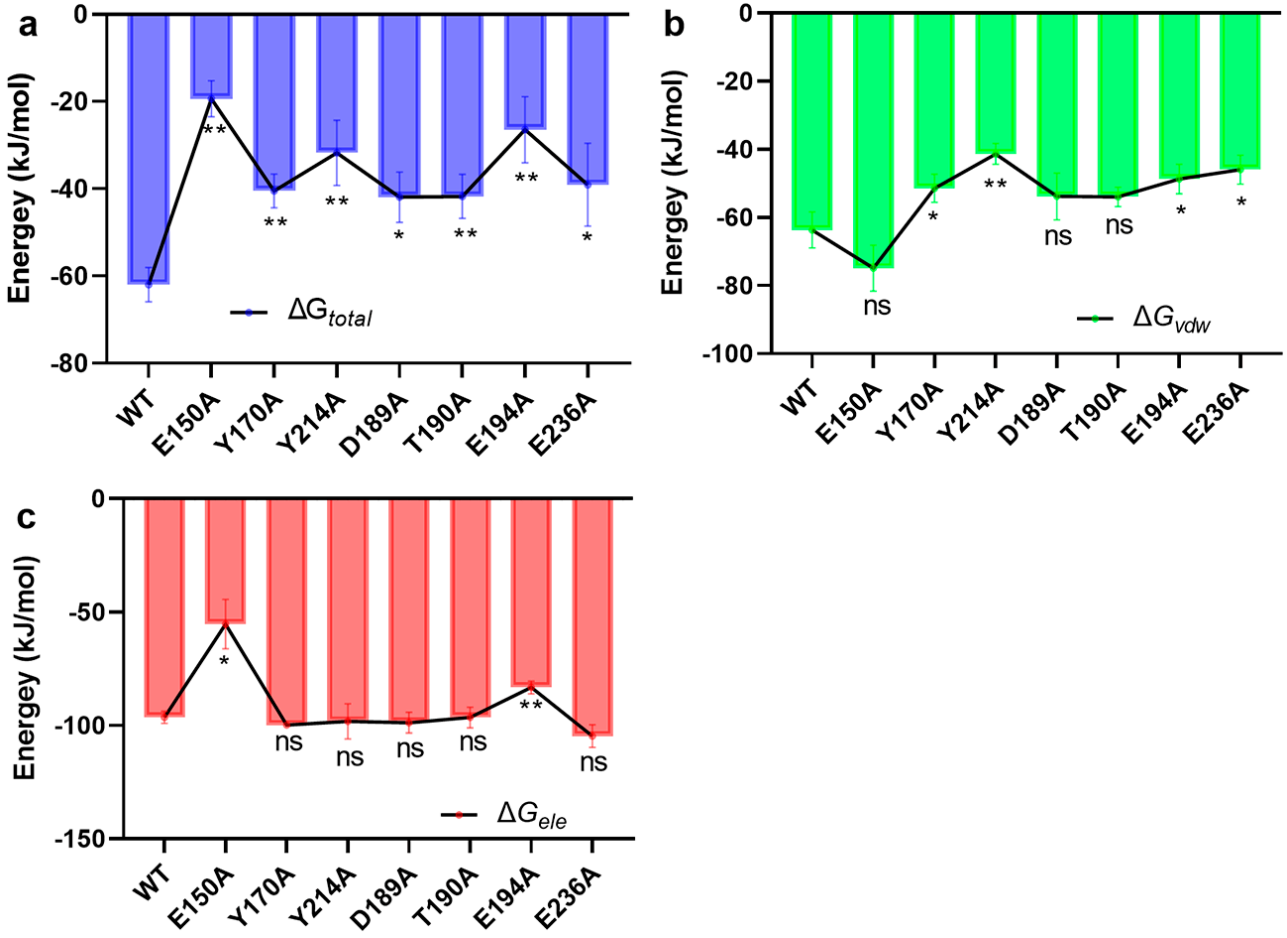
The binding free energy between Luteolin and inlB, or its mutants. The total binding free energy (**a**), the van der Waals interaction (**b**), and the electrostatic interactions (**c**). Data are presented as means ± SDs, n = 3, ns means no significant, * indicating *p* < 0.05, ** indicating *p* < 0.01.

To confirm the critical interactive residues, site-directed mutagenesis assays were performed and the binding free energies between the mutants and Luteolin were analyzed. The total binding free energy of all the mutants (E150A, Y170A, Y214A, D189A, T190A, E194A, and E236A) with Luteolin was significantly lower than that of the wild type protein (WT) (Figure 7a). Among them, the van der Waals interaction of Y170A, Y214A, E194A, and E236A with Luteolin was significantly lower than that of the WT protein (Figure 7b), which was consistent with the van der Waals energy contribution of these residues. The electrostatic interactions between Luteolin and E150A or E194A were significantly lower than the WT protein (Figure 7c). Glu150 and Glu194 contributed more electrostatic interactions, so the mutation of these residues resulted in a reduction in electrostatic interactions. The above results indicate that these residues are important for the binding of Luteolin to inlB.

#### 2.1.5. Luteolin Protects *G. mellonella* from LM Infection

**Figure 8:**
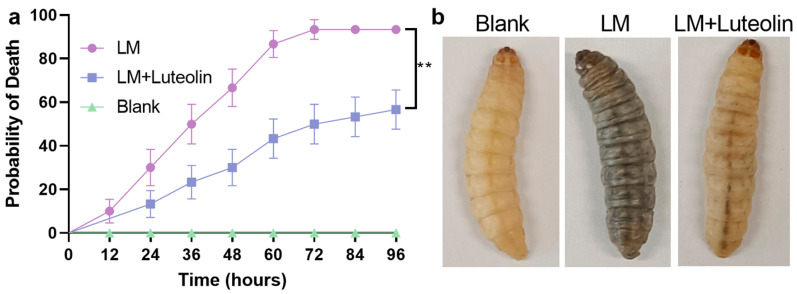
Luteolin protects *G. mellonella* from LM infection. (**a**) The probability of death of *G. mellonella* from different treatment groups. ** indicating *p* < 0.01. The survival rate analysis was based on the Log-rank (Mantel–Cox) test. (**b**) The symptoms of tissue pathological damage of samples from each group.

To evaluate the protective effect of Luteolin against LM infection in vivo, a *G. mellonella* infection model was established. By monitoring the survival of the samples, we found that there was no dead *G. mellonella* in the blank group until 72 h post-infection; the LM-infected group exhibited mortality within 12 h post-infection, with mortality rates gradually increasing until reaching 93.33% at 72 h. When Luteolin was applied to LM-infected samples, the dead *G. mellonella* was detected at 24 h, showing a slow upward trend until reaching 56.67% (Figure 8a). *G. mellonella* from the blank group shows normal state and posture, but the body of the LM-infected *G. mellonella* was swollen and filled with melanin; however, Luteolin treatment significantly alleviated both melanin formation and swelling severity (Figure 8b). These results demonstrate that Luteolin reduces mortality, improves survival rates, and mitigates pathological tissue symptoms of LM-infected *G. mellonella*.

## 3. Discussion

SrtA is the most important member of the sortase family that is expressed by Gram-positive pathogenic bacteria and has been the subject of the most thorough mechanism study [29]. *Staphylococcus aureus*, LM, *Streptococcus agalactiae*, *Streptococcus suis*, and some other Gram-positive bacteria can express SrtA; this protein mediates multiple pathogenic mechanisms, such as bacterial adhesion, invasion, biofilm formation, and persister bacteria formation. All these functions were related to the surface virulence proteins anchored by it [30,31]. *S. aureus* SrtA has been studied adequately, with as many as six resolved crystal structures (with or without natural ligands). The only crystal structure of LM SrtA was resolved in 2016, and His127, Arg197, and Cys188 were identified as its active sites; mutation of these three residues results in almost complete loss of the transpeptidation function of SrtA [27]. Reports of *S. aureus* SrtA inhibitors are plentiful, but inhibitors focused on LM SrtA are scarce. Chalcone and phloretin have been identified as LM SrtA inhibitors; chalcone interacted with Cys188 and Arg197 by generating Van der Waals interaction, while phloretin mainly interacted with Thr90, Val129, and Ile103 [27,32]. Here, Luteolin generated weak interaction (electrostatic and Van der Waals) with Cys188, Arg197, and His127, which are the active site residues of SrtA. These results suggest that Luteolin and chalcone share similar interactive mechanisms, as they all interacted with the active residues directly. Luteolin did not affect the structure of SrtA, as the Rg values were similar before and after binding with Luteolin; the RMSF changes in the residues also confirm these interactions. These three compounds (chalcone, phloretin, and luteolin) show similar IC_50_ values (28.41 µM, 37.24 µM, and 37.47 µM) against SrtA, among which phloretin and Luteolin are closer, as they share more similar structures. This information could provide important information for their application in the future.

Many LM internalin family members contain the LPXTG motif and can be anchored to cytoderm covalently by SrtA, thereby contributing their roles to promote LM infection [33]. InlA is such a LPXTG protein; its role mainly focuses on promoting LM adhesion and invasion into host cells [34]. In this study, we found that Luteolin, a natural flavonoid compound, likely binds to the active pocket of LM SrtA and forms weak interactions with the key active site residues His127, Arg197, and Cys188, resulting in reduced transpeptidation activity. This is confirmed by the enzyme kinetics experiment, as the Vmax did not show significant changes, but the Km increased significantly. SrtA is important for promoting LM adhesion and invasion by anchoring inlA to the cell wall; therefore, the inhibition of Luteolin against its activity may directly result in a reduction in inlA anchored on the cell wall, which decreases the adhesion and the invasion of LM to host cells.

InlB does not contain an LPXTG motif; it was anchored to the cell via non-covalent bonds, and binds to host receptors to help LM invade to host cells [35]. The crystal structure of the LRR domain (the smallest unit that binds to the host receptor) of inlB with the IG1 domain of its host receptor c-Met protein has been disclosed; the interacting residues include Asp128, Glu150, Tyr170, Tyr214, and Trp124 [36]. VHH, a nanobody derived from camel heavy-chain antibodies, has been reported to inhibit the invasion of LM by competitively binding to inlB [36]. Therefore, considering that inlB also plays a role in the adhesion and invasion of LM, we tried to explore whether an interaction existed between Luteolin and inlB; it was found that Luteolin binds to the groove of inlB where the interface of ilnB interacts with c-Met. It forms Hydrogen bonds with Glu150 and van der Waals interactions with Asp128, Tyr170, Tyr214, and Trp124, all of which are critical residues for the interaction between inlB and c-Met. The binding of Luteolin did not affect the structure of inlB, as it has similar Rg values with or without Luteolin. The smaller RMSF values of residues that interacted with Luteolin also confirm the binding. The binding free energies decreased significantly when these residues were mutated. Therefore, it was confirmed that Luteolin interacts with the critical residues involved in the inlB–Met interaction, which may affect their binding and subsequently impair the adhesion and invasion of LM to host cells.

Luteolin has been reported to reduce the transcription of LLO by directly binding to the coding region of *hly* mRNA, thereby decreasing the expression and secretion of LLO into the extracellular environment [26]. As the “Swiss Army knife” of LM, LLO also plays a pivotal role in LM infection and is widely recognized as a key virulence factor for LM infection [37,38]. This study confirmed that besides LLO, Luteolin inhibited the adhesion and invasion of LM by targeting SrtA and inlB. More importantly, by establishing a LM-infected *G. mellonella* model and treating them with Luteolin, we found that Luteolin significantly improved the survival rate of LM-infected *G. mellonella* and alleviated tissue damage symptoms. The protective effect of Luteolin against LM infection in vivo models originates from its simultaneous inhibitory effects on LLO, SrtA, and inlB. Of course, there may still be other potential undiscovered mechanisms.

## 4. Materials and Methods

### 4.1. Protein, Cells, Strains, and Reagents

Plasmid (pET-28a) that inserted *SrtA* gene was stored in our laboratory; the protein was purified based on a method described previously [32]. Human colorectal adenocarcinoma cells, Caco2 cells, were obtained from American Type Culture Collection (ATCC); cells were cultured in Dulbecco’s modified Eagle’s medium (DMEM, Sangon Biotech, Shanghai, China) with 10% fetal bovine serum (FBS, Sangon Biotech, Shanghai, China). LM ATCC BAA-679 strain was purchased from ATCC (Manassas, VA, USA), the bacteria was cultured in Brain Heart Infusion (BHI, Hopebiol, Qingdao, China) Broth without or with agar.

### 4.2. SrtA Activity Inhibition Assay and the Kinetics

Purified SrtA protein was mixed with Luteolin at different concentrations (0, 28, 56, 112 µM) in black 96-well plates and co-incubated at 37 °C for 30 min. Subsequently, the substrate peptide with fluorophores conjugated (Dabcyl-QALPETGEE-Edans, GL Biochem, Shanghai, China) to both ends was added, and the mixture was further incubated in the dark for 60 min. The fluorescence intensity was then measured (excitation wavelength: 350 nm; emission wavelength: 520 nm), and the biological activity of SrtA under different treatment conditions was analyzed [32]. To determine the inhibitory mechanism, an enzymatic kinetics test was performed. Specifically, samples that contain the protein and substrate peptide were used to detect the fluorescence signal every ten minutes to determine the reaction process. After this, SrtA was treated with or without Luteolin (112 µM), and then different concentrations (0.6 µM, 3 µM) of substrate peptide were added to the specific well; after co-incubation, samples were used to detect the fluorescence signal. The reaction rate was obtained as follows: the increment of the fluorescence signal divided by the reaction time. Then, the Linweaver–Burk was performed to obtain the relative Vmax and Km. Protease K (TIANGEN, Beijing, China) was used as a standard to ensure all the substrate were split, while samples that did not contain substrates were used as negative control.

### 4.3. Molecular Docking

The crystal structures of LM SrtA (PDB ID: 5HU4) and inlB (PDB ID: 1D0B) were obtained from the PDB database and they were used as receptors. Before docking, the proteins were used to perform a 50 ns MD assay to harvest a stable structural model. The structure of Luteolin (CAS Number: 491-70-3) was obtained from PubChem and it was used as ligand. Prior to docking, Luteolin and the proteins were processed using Autodock Tools 1.5.6 version. The sizes of the docking boxes for Luteolin and SrtA or inlB were 48 × 46 × 40 Å and 40 × 58 × 52 Å, respectively, with a grid spacing of 1 Å. The docking calculation was performed using Autodock Vina (v1.2.7) [39]; the binding between Luteolin and SrtA or inlB were analyzed based on the affinity scores.

### 4.4. Molecular Simulation

The complexes of Luteolin with SrtA or inlB that obtained from the docking assay were used as the initial configurations to perform the MD simulation assay by referring to a previously reported method [40]. GROMACS [41] 2020.6 version was applied. The core parameters are as follows: amber99SB-ILDN force field and TIP3P water mode were used. The simulation process is mainly as follows: a box was generated with the complex in the center; then, the solvent and counter ions were added. After energy minimization, the system reached a constant-pressure and constant-temperature condition following an NPT MD; then, a 50 ns MD assay was performed. After the completion of the simulation, the RMSD and distances between Luteolin and the target proteins were analyzed to evaluate the equilibrium and binding stability of the systems. The Rg was analyzed to evaluate the structure changes in the proteins when bound with or without Luteolin. The Molecular Mechanics/Poisson–Boltzmann Surface Area (MMPBSA) method [42,43] was employed to analyze the binding free energies of the Luteolin–SrtA and Luteolin–inlB complexes, as well as the contribution of each residue to the binding free energy. The RMSF was detected to determine the flexibility of the residues that interacted with Luteolin. To confirm the critical residues that involved the binding of inlB and Luteolin, the mutants (E150A, Y170A, Y214A, D189A, T190A, E194A, E236A) of inlB were generated by using Swiss PDB Viewer software v4.1 [44] to calculate the binding free energy.

### 4.5. Minimum Inhibitory Concentration (MIC) and Growth Curve Assay

The MIC of Luteolin against LM was determined based on the method described in Clinical and Laboratory Standards Institute (CLSI). Briefly, serious concentrations of Luteolin (0–448 µM) were prepared in BHI medium, then the bacteria were added to reach a final concentration of 5 × 10^5^ CFUs/mL. Samples were cultured at 37 °C for 24 h. The minimum concentration that did not grow bacteria was defined as the MIC value. For the growth curve assay, LM was cultured in BHI without or with Luteolin (112 µM) at 37 °C with shaking. The initial density of LM was approximately 3.0 × 10^8^ CFUs/mL; samples were harvested every one hour to measure the absorbance value at 600 nm (Abs_600_); the growth curves were plotted to analyze the effect of Luteolin on the growth of LM [26]. For both of the MIC and growth curve assays, Gentamicin was used as a standard for control.

### 4.6. Cytotoxicity Detection and Cell Adhesion Assay

The cytotoxicity of Luteolin on Caco2 was determined by using an LDH kit (Beyotime, Shanghai, China). A sheep red blood cell hemolysis assay was performed to visualize the cytotoxicity of Luteolin. Sterile PBS containing sterile de-fibrinated sheep red blood cells (2.5%) were added to various concentrations of Luteolin and co-incubated for thirty minutes; the images were obtained after samples were centrifuged. For adhesion assay, Caco2 cells were seeded into 24-well plates at a density of 2 × 10^5^ cells/well and cultured overnight. LM was pre-treated with different concentrations of Luteolin (0, 56, 112 µM), after co-culturing until the logarithmic phase; the bacteria were harvested and suspended in DMEM medium to replace the cell culture medium; Luteolin at different concentrations was added. The multiplicity of infection (MOI) was 100. Samples were co-cultured for one hour, then the medium was removed and the cells were washed three times with sterile phosphate saline buffer (PBS); cells were harvested and diluted. The samples were spread onto BHI agar plates. Following overnight incubation, the number of bacterial colonies was counted to analyze the effect of Luteolin on bacterial adhesion to the cells [45].

### 4.7. Cell Invasion Assay

Caco2 cells were seeded into 24-well plates (2 × 10^5^ cells/well) and cultured overnight. The next day, DMEM suspended with LM (pre-treated with Luteolin) and different concentrations of Luteolin (0, 56, 112 µM) was used to treat the cells with a MOI of 100. After co-cultured for 1.5 h, the cultural medium was replaced with free DMEM that contained gentamicin (170 µM). After one hour of treatment, the medium was discarded, and the cells were lysed with 0.2% saponin after being washed. Equal volume of sample was spread onto BHI agar medium and cultured overnight. The next day, the number of bacterial colonies was counted to analyze the inhibitory effect of Luteolin on the invasion of LM [32].

### 4.8. Infection Model

Logarithmic growth phase LM was harvested and suspended to sterile PBS. The bacteria (10 µL) were injected into *G. mellonella* (1 × 10^6^ CFUs/sample) via a micro-injection pump. A total of twenty infected samples were randomly divided into two groups, with 10 samples per group. One group was injected with Luteolin (105 µmol/kg) as the treatment group; the other group was injected with an equal volume of solvent and recorded as the infected group, and the blank control group was injected with an equal volume of sterile PBS. The survival status of *G. mellonella* was observed every twelve hours, and the survival rate was calculated to analyze the protective effect of Luteolin against LM infection on *G. mellonella*. Samples were collected from different treatment groups for observation to evaluate the alleviating effect of Luteolin on the symptoms of LM-infected *G. mellonella* [46,47].

### 4.9. Statistical Analysis

Experimental data from three independent replicate experiments are presented as mean ± standard deviations (SDs). Statistical analysis was performed using the unpaired *t*-test that implanted into GraphPad Prism 9.5.0 software. Survival rate analysis was based on the Log-rank (Mantel–Cox) test. The results were considered statistically significant when *p* < 0.05.

## 5. Conclusions

Luteolin, a natural flavonoid compound, exhibits no antibacterial activity at the tested concentrations. It binds to LM SrtA and inlB, which may reduce the anchor of inlA to the cell wall mediated by SrtA, and may affect the binding between inlB and its host receptor; thereby, the adhesion and invasion of LM to host cells were reduced. Furthermore, Luteolin reduced the mortality of *G. mellonella* infected with LM and alleviated the infection symptoms. Combined with previous reports that Luteolin inhibits the expression and secretion of LLO, these findings indicate that Luteolin exerts inhibitory effects on the functions of multiple critical virulence factors of LM infection. The results of this study provide more clear and convincing evidence for the application of Luteolin in the prevention and control of LM infection, which is expected to promote its application in the future.

## Data Availability

Data is provided within the manuscript.

## Supplementary Materials

The following supporting information can be downloaded at: https://www.mdpi.com/article/10.3390/molecules31020297/s1, Figure S1. The RMSF values of SrtA when bound with or without Luteolin. Figure S2. The reaction process of SrtA (a) and the fitted regression lines based on Linweaver-Burk (b). Figure S3. The cytotoxicity of Luteolin. The death of the Caco2 cells (a) and the images of the sterile defibrated sheep red blood cells (b) when treated with various concentrations of Luteolin. Figure S4. The superposition of Luteolin-inlB complex to inlB-receptor complex. Figure S5. The Rg (a) and the RMSF (b) fluctuation of inlB when bound with or without Luteolin. Table S1. The relative values of Vmax and Km of SrtA when treated with or without Luteolin. Table S2. The MIC values of Luteolin against LM.
