# Supplementary material for: Luteolin Inhibits Invasion of Listeria monocytogenes by Interacting with SortaseA and InternalinB
Source: Molecules. 2026 Jan 14;31(2):297. doi: 10.3390/molecules31020297 (PMC12844107; doi:10.3390/molecules31020297)
Supplement: Supplementary file 1 [file molecules-31-00297-s001.zip › molecules-4044708-supplementary.pdf]

Supplementary files for

Luteolin Inhibits Invasion of Listeria monocytogenes by Interacting with SortaseA and InternalinB

Junlu Liu [1,†], Rui Liu [1,†], Hang Pan [1], Jiahui Lu [1], Qiong Liu [1,2] and Guizhen Wang [1,*]

1 College of Biological and Food Engineering, Jilin Engineering Normal University, Changchun 130052, China; liujunlu@stu.jlenu.edu.cn (J.L.); liurui@stu.jlenu.edu.cn (R.L.); panhang@stu.jlenu.edu.cn (H.P.); lujiahui@stu.jlenu.edu.cn (J.L.); liuqiong@jlenu.edu.cn (Q.L.)

2 Engineering Research Center of Microecological Vaccines (Drugs) for Major Animal Diseases, Ministry of Education, Jilin Agricultural University, Changchun 130118, China

* Correspondence: wanggz@jlenu.edu.cn

† These authors contributed equally to this work.

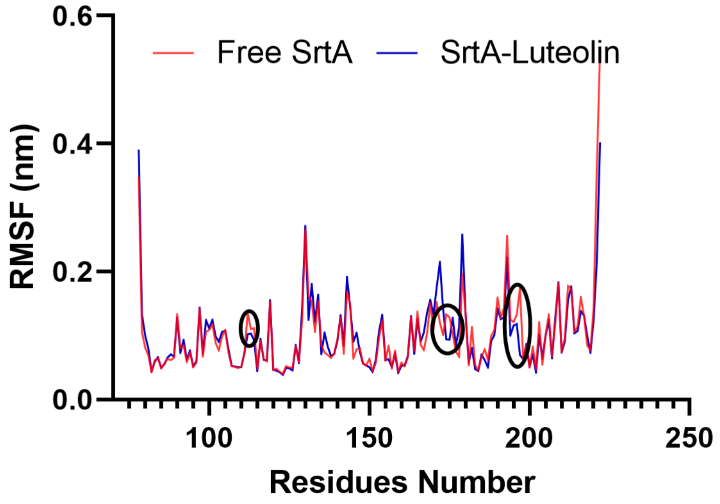

Figure S1 The RMSF values of SrtA when bound with or without Luteolin.

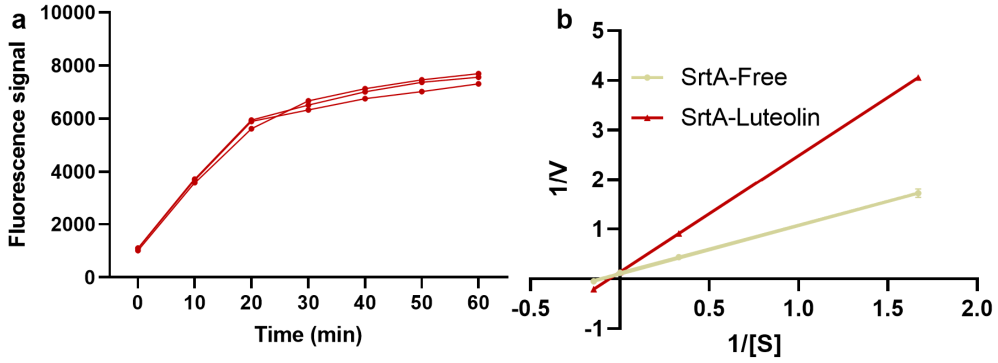

Figure S2 The reaction process of SrtA (a) and the fitted regression lines based on Linweaver-Burk (b).

Table S1 The relative values of Vmax and Km of SrtA when treated with or without Luteolin

Index	SrtA-Free	SrtA-Luteolin	P values	Significance
V_{max} (×100)	8.27 ± 1.22	7.35 ± 0.48	0.40	ns
K_m	8.00 ± 1.63	17.25 ± 1.22	0.0039	**

Note: The values of V_{max} and K_m are relative based on the changes of the fluorescence signal, they are not the real values of the protein. The ns means no significant, ** represents $p<0.01$.

Table S2 The MIC values of Luteolin against LM

Compound	MIC (μM)
Luteolin	448
Gentamicin	3.4

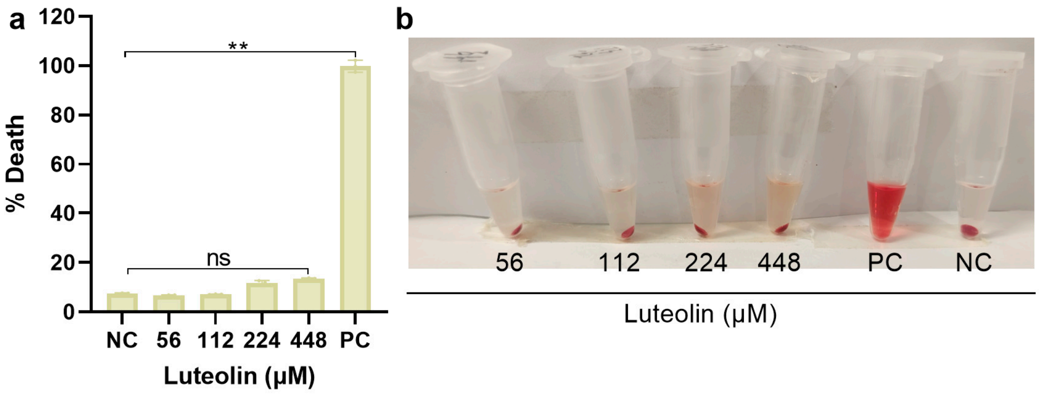

Figure S3 The cytotoxicity of Luteolin. The death of the Caco2 cells (a) and the images of the sterile defibrated sheep red blood cells (b) when treated with various concentrations of Luteolin. Data were shown as means with SDs, n=3, ns indicates no significant, ** represents $p<0.01$.

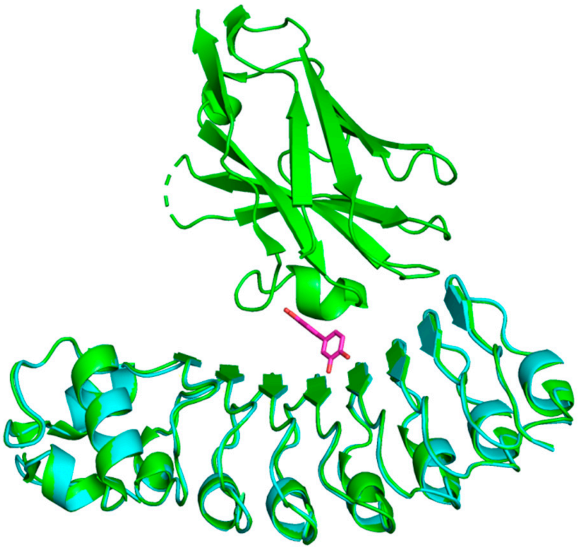

Figure S4 The superposition of Luteolin-inlB complex to inlB-receptor complex. Luteolin was shown as stick and colored by element, the proteins were shown as secondary structure and colored by chain. InlB-receptor complex was colored by green, inlB bound with Luteolin was colored by cyan.

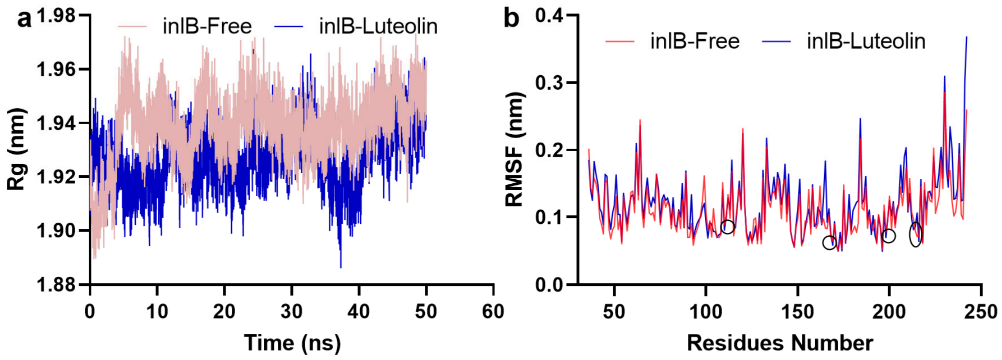

Figure S5 The Rg (a) and the RMSF (b) fluctuation of inlB when bound with or without Luteolin.
